# Supplementary material for: Angular difference in human coronary artery governs endothelial cell structure and function
Source: Commun Biol. 2022 Oct 1;5:1044. doi: 10.1038/s42003-022-04014-3 (PMC9526720; doi:10.1038/s42003-022-04014-3)
Supplement: Supplementary file 2 — Supplemental Information [file 42003_2022_4014_MOESM2_ESM.pdf]

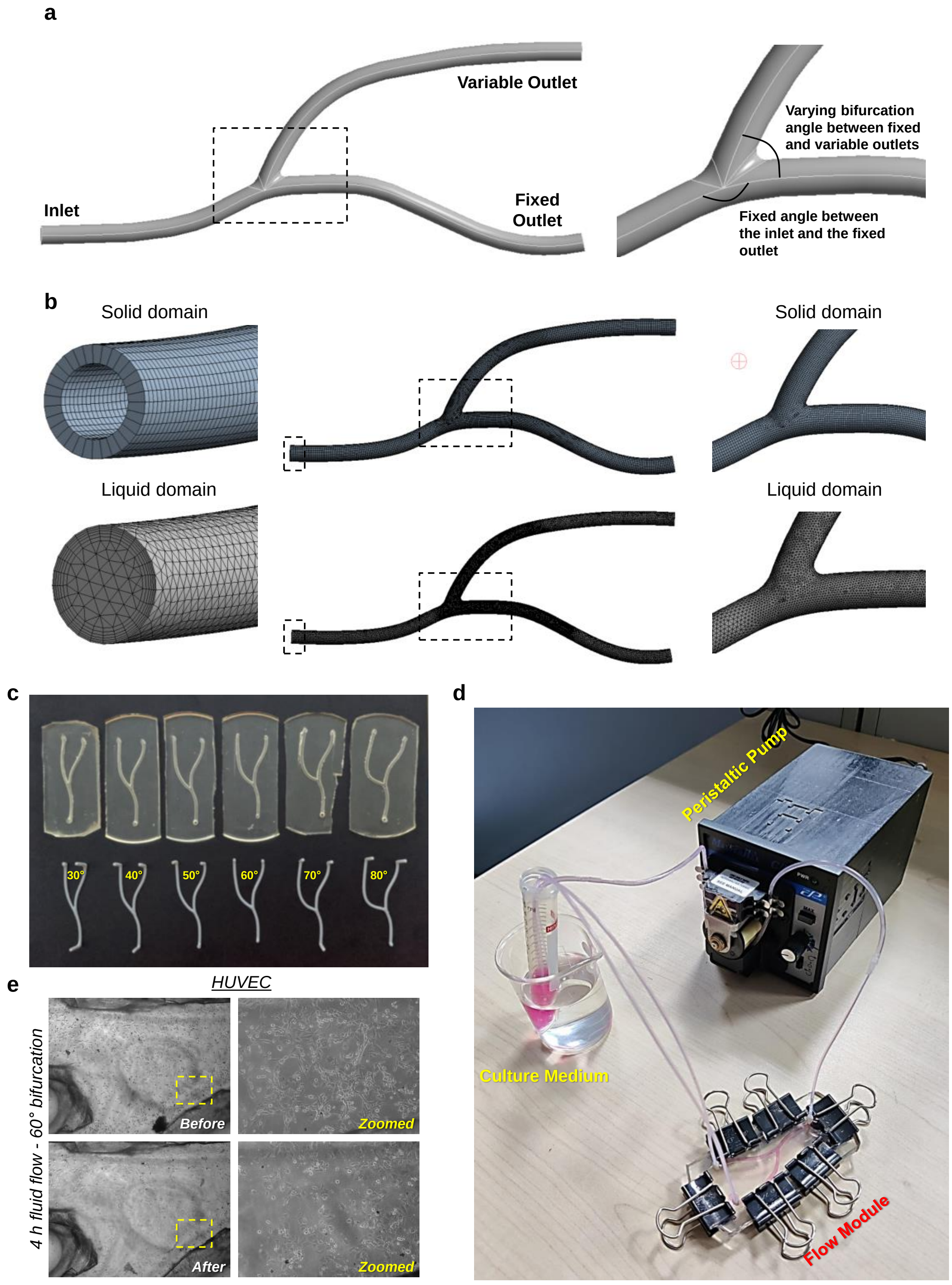

Supplemental Figure 1. Modelling, 3D-printing and preparation of microchannels with varying angles of bifurcations. (a) The geometry of the right coronary artery with a 60° bifurcation angle etween the two outlets, keeping the angle between the inlet and the fixed-outlet constant. (b) A representative mesh

of the geometry of 60° ifurcation angle with 42,000 quadrilateral elements in the solid domain and 489119 tetrahedral elements in the fluid domain. (c) 3D-printed branched coronary arteries with bifurcation angles of 30°, 40°, 50°, 60°, 70°, and 80° (bottom) and their respective PDMS microchannels (top). (d) *In vitro* fluid flow exposure setup consisting of the culture medium reservoir, peristaltic pump and the flow module or the microchannel. (e) Bright field images of HUVEC pre- and post-4 h flow exposure in the 60° angled flow module.

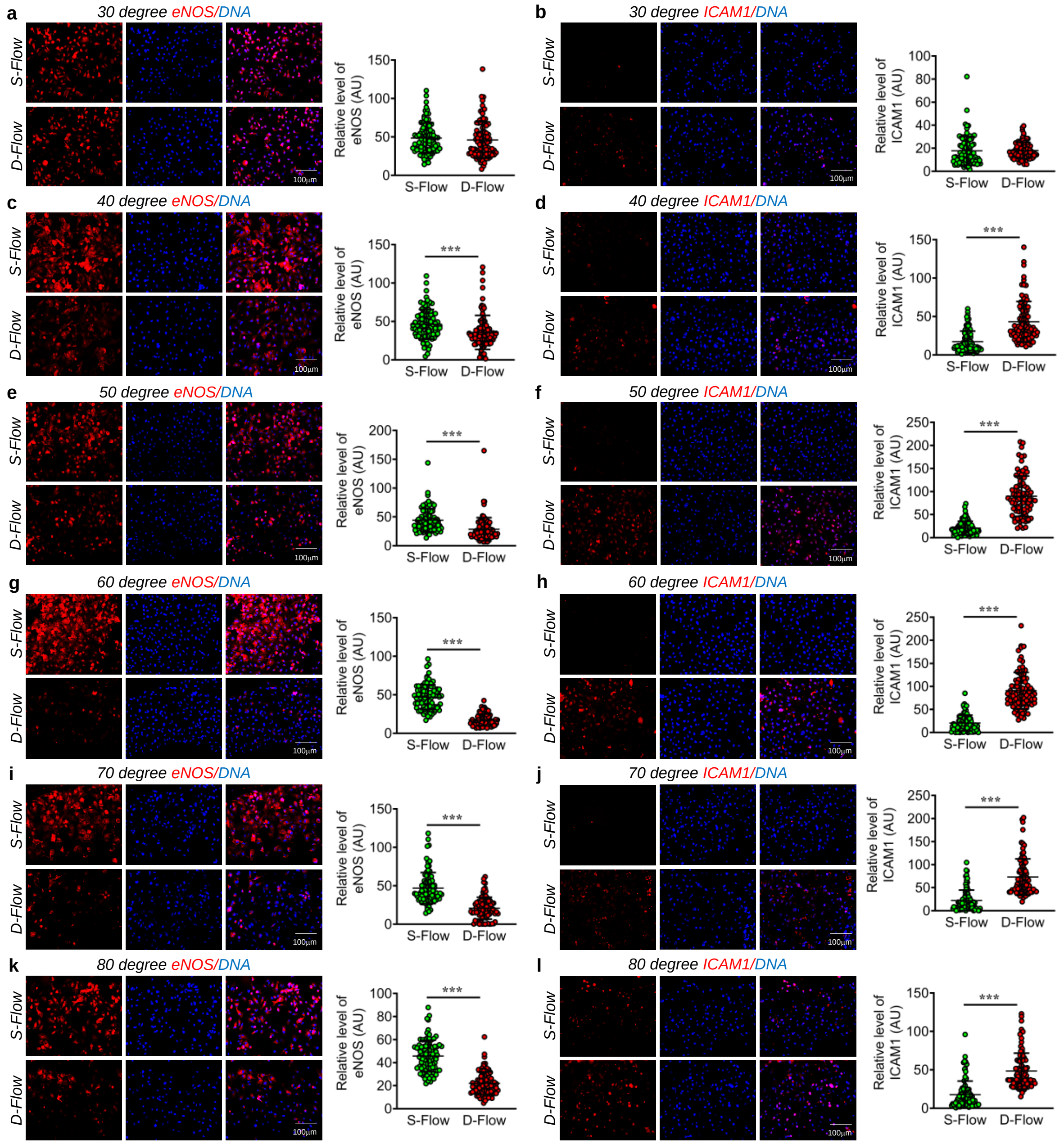

Supplemental Figure 2. Microchannels with varying angles of bifurcation have differential effects on EC biochemical signatures. (a-l) Immunofluorescence staining of EA.hy926 for eNOS (n = 3) and ICAM1 (n = 3) after 4 h fluid flow exposure, using a human right-coronary artery based microchannel with a bifurcation angle of 30° (a,b), 40° (c,d), 50° (e,f), 60° (g,h), 70° (i,j), and 80° (k,l). DAPI staining is shown in blue. eNOS and ICAM1 fluorescence signal in individual EA.hy926 cells (dots) from three individual experiments. Fluorescence intensity AU values per individual cells are indicated together with the mean.

Total number of cells, $n \geq 90$. Values represent the mean ± SD. *$p < 0.05$, **$p < 0.01$, and ***$p < 0.001$, by unpaired *t* test. Magnification: 20x, Scale: 100μm.

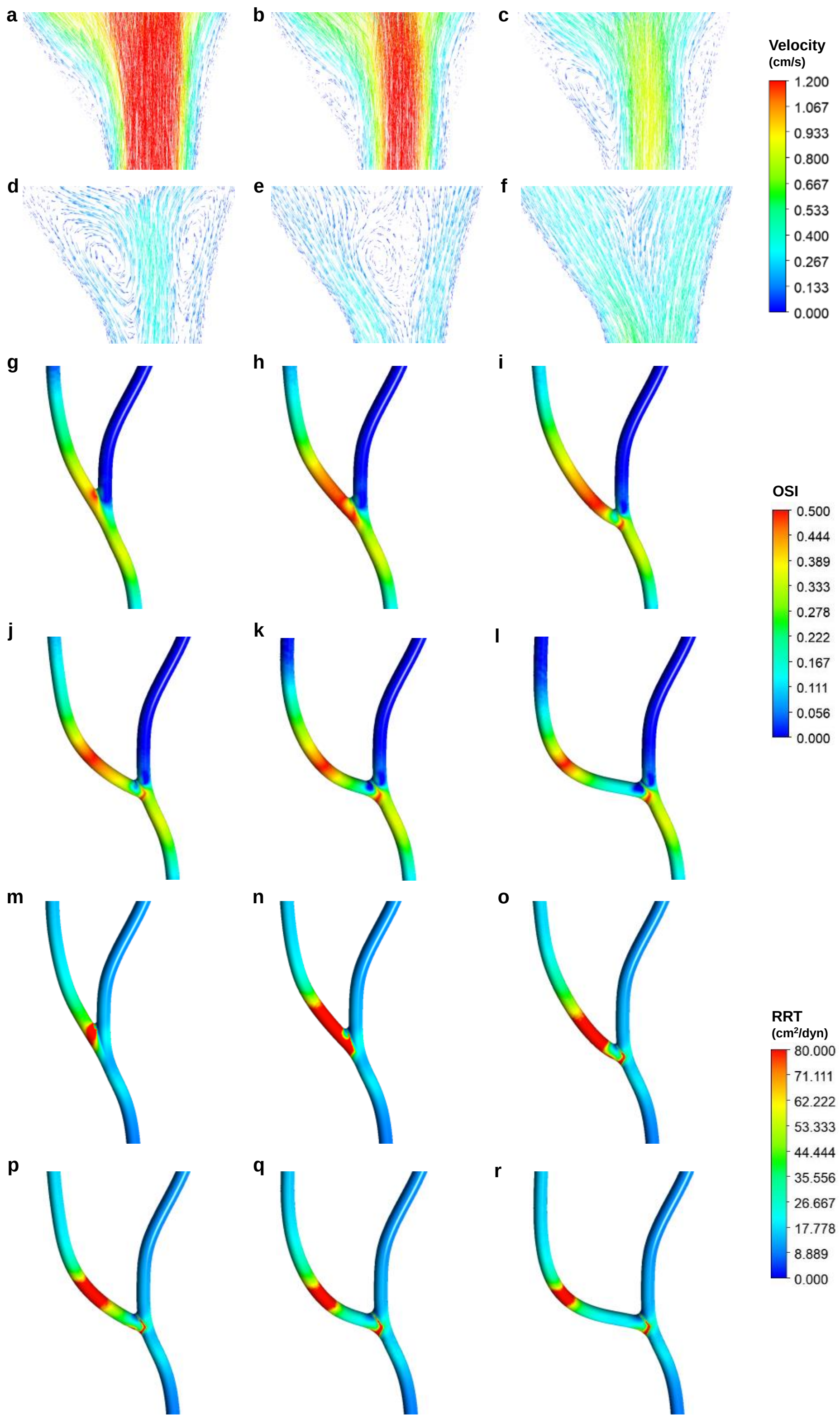

Supplemental Figure 3. Pulsatile simulation induces re-circulatory flow patterns at the bifurcation and elevates oscillatory shear and residence time across the vessel. (a-f) Representative velocity vectors

in the pulsatile studies of 60° bifurcation angle at t = 0.41 s (a), t = 0.415 s (b), t = 0.42 s (c), t = 0.425 s (d), t = 0.43 s (e), and t = 0.435 s (f) in the bifurcation region. (g-l) OSI contours of pulsatile-rigid studies in vessels with 30° (g), 40° (h), 50° (i), 60° (j), 70° (k), and 80° (l) bifurcation angles. (m-r) RRT contours of pulsatile-rigid studies using 30° (m), 40° (n), 50° (o), 60° (p), 70° (q), and 80° (r) bifurcation angles.

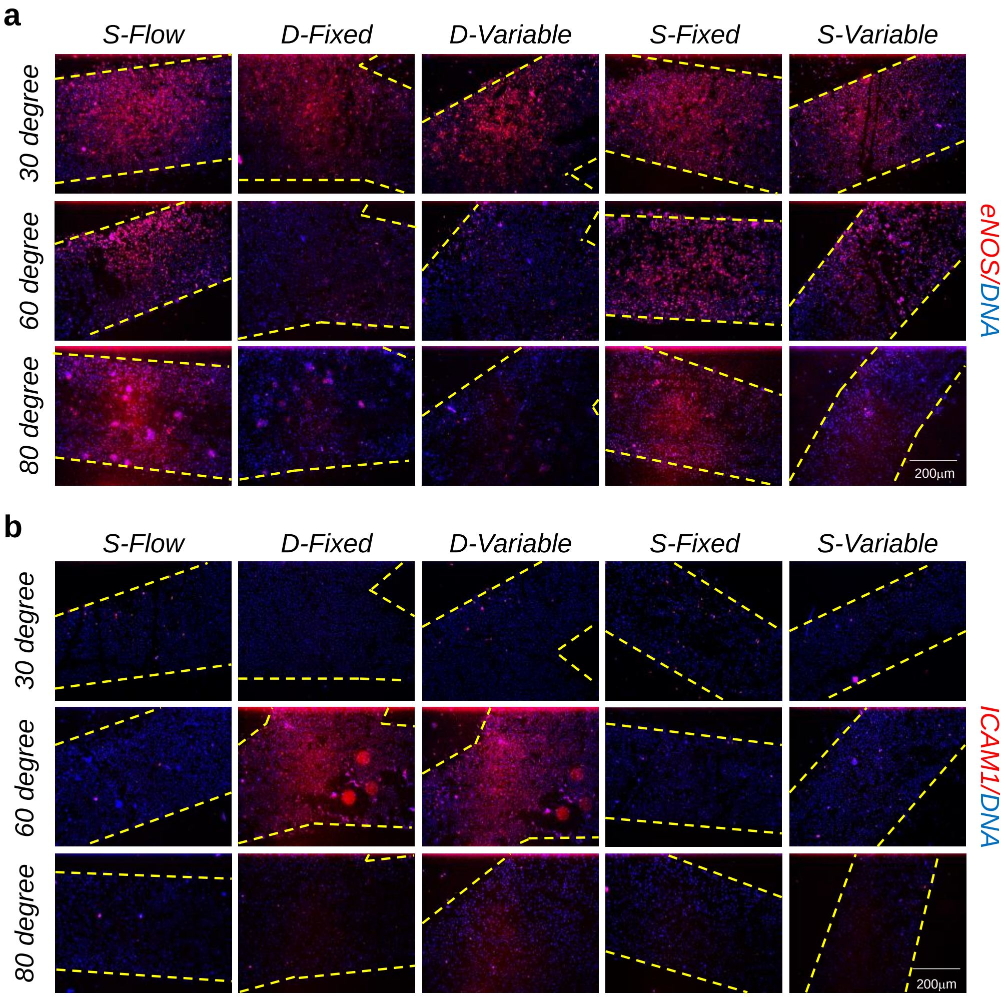

Supplemental Figure 4. Varying angles of vessel bifurcation differentially lower eNOS and increase ICAM1 protein levels, *in vitro*. (a-b) Immunofluorescence staining of EA.hy926 for eNOS (a, *n* = 3) and ICAM1 (b, *n* = 3) after 4 h fluid flow exposure, using a human right-coronary artery based microchannel with a bifurcation angle of 30°, 60°, and 80°. DAPI staining is shown in blue. Magnification: 5x, Scale: 200µm.

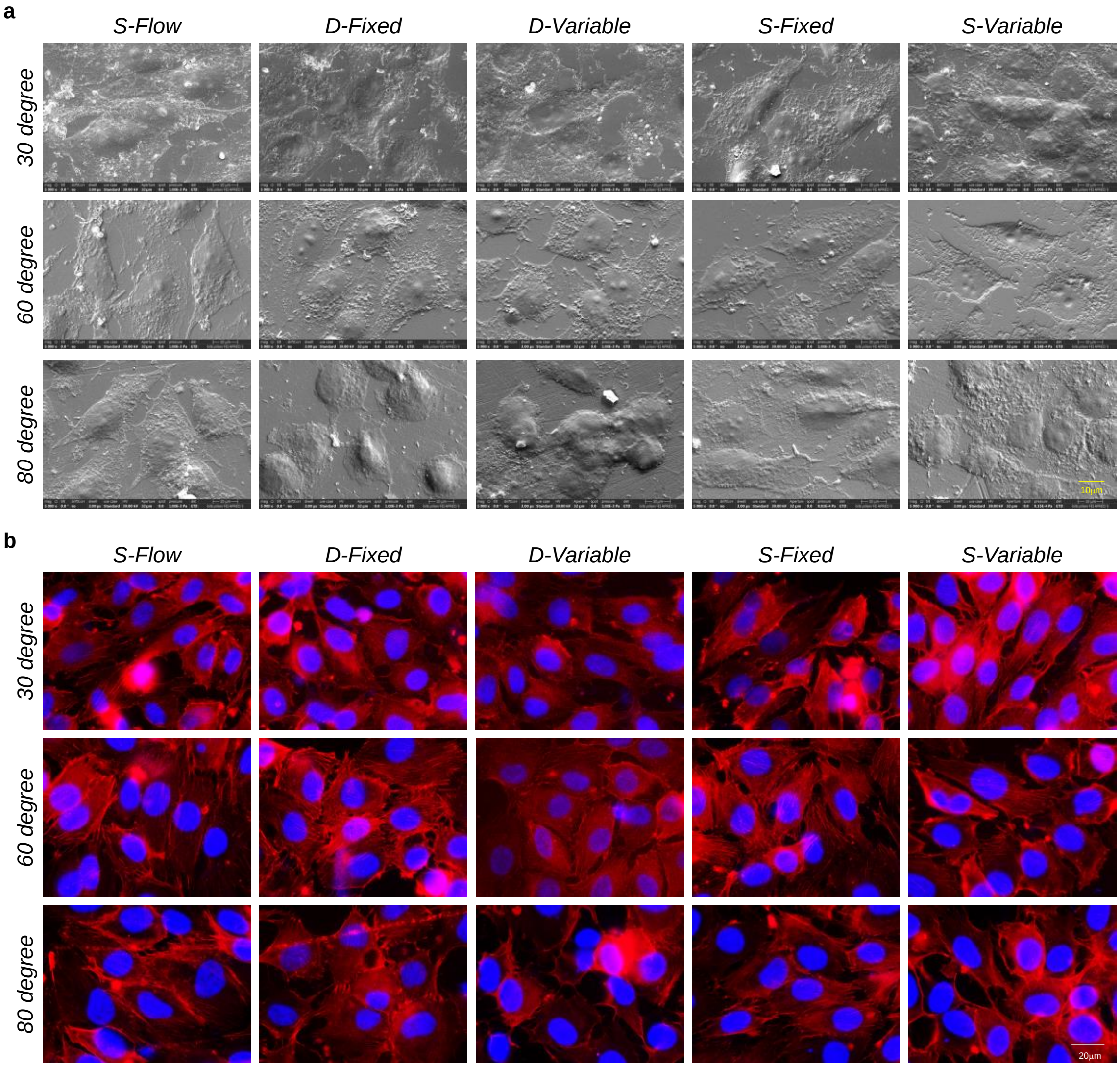

Supplemental Figure 5. Increasing angular bifurcations affect EC morphology at different locations across the microchannel. (a-b) Area-specific morphometric assessment of EA.hy926 exposed to flow in microchannels with bifurcation angles of 30, 60, and 80° through FE-SEM (a) and F-actin staining (b). Magnification: 5000x/ 63x, Scale: 10µm/ 20µm.
