# Supplementary material for: Angular difference in human coronary artery governs endothelial cell structure and function
Source: Commun Biol. 2022 Oct 1;5:1044. doi: 10.1038/s42003-022-04014-3 (PMC9526720; doi:10.1038/s42003-022-04014-3)
Supplement: Supplementary file 3 — Description of Additional Supplementary Files [file 42003_2022_4014_MOESM3_ESM.pdf]

Description of Additional Supplementary Files

File name: Supplemental Data 1
Description: The source data behind the graphs in the paper.

File name: Supplemental Movie 1
Description: Recording of the magnetic beads flowing through the S-Flow region of the microchannel.

File name: Supplemental Movie 2
Description: Recording of the magnetic beads flowing through the D-Fixed region of the 30 degree microchannel.

File name: Supplemental Movie 3
Description: Recording of the magnetic beads flowing through the D-Variable region of the 30 degree microchannel.

File name: Supplemental Movie 4
Description: Recording of the magnetic beads flowing through the D-Fixed region of the 60 degree microchannel.

File name: Supplemental Movie 5
Description: Recording of the magnetic beads flowing through the D-Variable region of the 60 degree microchannel.

File name: Supplemental Movie 6
Description: Recording of the magnetic beads flowing through the D-Fixed region of the 80 degree microchannel.

File name: Supplemental Movie 7
Description: Recording of the magnetic beads flowing through the D-Variable region of the 80 degree microchannel.
