# Supplementary material for: Angular difference in human coronary artery governs endothelial cell structure and function
Source: Commun Biol. 2022 Oct 1;5:1044. doi: 10.1038/s42003-022-04014-3 (PMC9526720; doi:10.1038/s42003-022-04014-3)
Supplement: Supplementary file 12 — Reporting summary [file 42003_2022_4014_MOESM12_ESM.pdf]

nature portfolio

Corresponding author(s): Dr. Syamantak Majumder and Dr. Bhanu Vardhan Reddy Kuncharam

Last updated by author(s): Sep 13, 2022

Reporting Summary

Nature Portfolio wishes to improve the reproducibility of the work that we publish. This form provides structure for consistency and transparency in reporting. For further information on Nature Portfolio policies, see our Editorial Policies and the Editorial Policy Checklist.

Statistics

For all statistical analyses, confirm that the following items are present in the figure legend, table legend, main text, or Methods section.

n/a	Confirmed	
☐	☒	The exact sample size (*n*) for each experimental group/condition, given as a discrete number and unit of measurement
☐	☒	A statement on whether measurements were taken from distinct samples or whether the same sample was measured repeatedly
☐	☒	The statistical test(s) used AND whether they are one- or two-sided<br>*Only common tests should be described solely by name; describe more complex techniques in the Methods section.*
☐	☒	A description of all covariates tested
☐	☒	A description of any assumptions or corrections, such as tests of normality and adjustment for multiple comparisons
☐	☒	A full description of the statistical parameters including central tendency (e.g. means) or other basic estimates (e.g. regression coefficient) AND variation (e.g. standard deviation) or associated estimates of uncertainty (e.g. confidence intervals)
☐	☒	For null hypothesis testing, the test statistic (e.g. *F*, *t*, *r*) with confidence intervals, effect sizes, degrees of freedom and *P* value noted<br>*Give P values as exact values whenever suitable.*
☒	☐	For Bayesian analysis, information on the choice of priors and Markov chain Monte Carlo settings
☒	☐	For hierarchical and complex designs, identification of the appropriate level for tests and full reporting of outcomes
☒	☐	Estimates of effect sizes (e.g. Cohen's *d*, Pearson's *r*), indicating how they were calculated

Our web collection on statistics for biologists contains articles on many of the points above.

Software and code

Policy information about availability of computer code

Data collection	ANSYS Fluent, version 17.2
Data analysis	ImageJ, GraphPad Prism

For manuscripts utilizing custom algorithms or software that are central to the research but not yet described in published literature, software must be made available to editors and reviewers. We strongly encourage code deposition in a community repository (e.g. GitHub). See the Nature Portfolio guidelines for submitting code & software for further information.

Data

Policy information about availability of data

All manuscripts must include a data availability statement. This statement should provide the following information, where applicable:

- Accession codes, unique identifiers, or web links for publicly available datasets
- A description of any restrictions on data availability
- For clinical datasets or third party data, please ensure that the statement adheres to our policy

All the original immunofluorescence analysis data generated and analyzed in this study are available in the supplementary section (Supplemental Raw data File). All other data can be obtained from the corresponding authors on reasonable request.

March 2021

Human research participants

Policy information about studies involving human research participants and Sex and Gender in Research.

Reporting on sex and gender	Not Applicable
Population characteristics	Not Applicable
Recruitment	Not Applicable
Ethics oversight	Not Applicable

Note that full information on the approval of the study protocol must also be provided in the manuscript.

Field-specific reporting

Please select the one below that is the best fit for your research. If you are not sure, read the appropriate sections before making your selection.

☒ Life sciences ☐ Behavioural & social sciences ☐ Ecological, evolutionary & environmental sciences

For a reference copy of the document with all sections, see nature.com/documents/nr-reporting-summary-flat.pdf

Life sciences study design

All studies must disclose on these points even when the disclosure is negative.

Sample size	Number of cells analyzed determined the sample size with the least being greater than or equal to 30.
Data exclusions	No data were excluded from the analyses.
Replication	All the findings reported here were successfully replicated atleast thrice or in certain cases five times. We faced no challenges in replicating the experimental setup and datasets.
Randomization	Cells from different passage numbers were used to obtain the datasets.
Blinding	All data were analyzed as a single blind undertaking.

Reporting for specific materials, systems and methods

We require information from authors about some types of materials, experimental systems and methods used in many studies. Here, indicate whether each material, system or method listed is relevant to your study. If you are not sure if a list item applies to your research, read the appropriate section before selecting a response.

Materials & experimental systems

n/a	Involved in the study
☐	☒ Antibodies
☐	☒ Eukaryotic cell lines
☒	☐ Palaeontology and archaeology
☒	☐ Animals and other organisms
☒	☐ Clinical data
☒	☐ Dual use research of concern

Methods

n/a	Involved in the study
☒	☐ ChIP-seq
☒	☐ Flow cytometry
☒	☐ MRI-based neuroimaging

Antibodies

Antibodies used	eNOS (D9A5L) Rabbit mAb (#32027; Lot 3), CD54/ICAM-1 Rabbit pAb (#4915; Lot 2) and CD31 (PECAM-1) (89C2) Mouse mAb (#3528; Lot 7) from Cell Signaling Technology, and CD144 (VE-Cadherin) Mouse mAb (#SC-9989, Lot G1421) from Santa Cruz Biotechnologies.
Validation	All the above mentioned antibodies have been tested and reported to be "Human" reactive by the manufacturer. The Cell Signaling Technology / Santa Cruz Biotechnologies' website adequately elaborates on the citations and other specifications of these antibodies (www.cellsignal.com/).

Eukaryotic cell lines

Policy information about cell lines and Sex and Gender in Research

Cell line source(s)	EA.hy926 were purchased from ATCC, The USA (#CRL-2922); HUVEC were procured from HiMedia Laboratories, Mumbai, India (#CL002).
Authentication	Detailed cell line authentication is available on the ATCC website (www.atcc.org) / HiMedia website (www.himedialabs.com).
Mycoplasma contamination	Cell supplied by ATCC and HiMedia Laboratories are tested for mycoplasma contamination. Both the cells procured for this study were mycoplasma negative.
Commonly misidentified lines (See ICLAC register)	None
